# STAT3 paradoxically stimulates β-catenin expression but inhibits β-catenin function

**DOI:** 10.1111/iep.12102

**Published:** 2014-10-28

**Authors:** Salih Ibrahem, Saleh Al-Ghamdi, Kanwal Baloch, Belal Muhammad, Wakkas Fadhil, Darryl Jackson, Abdolrahman S Nateri, Mohammad Ilyas

**Affiliations:** * Academic Unit of Molecular Pathology, Nottingham UniversityNottingham, UK; † Cancer Genetics & Stem Cell Group, Division of Cancer and Stem Cells, School of Medicine, Queen's Medical Centre, Nottingham UniversityNottingham, UK

**Keywords:** colorectal cancer, oncogene interaction, STAT3 signalling, Wnt signalling

## Abstract

Wnt signalling and the signal transducer and activator of transcription 3 (STAT3) are oncogenic signalling pathways which are deregulated in colorectal cancer (CRC). Here we investigated the interaction of these two pathways. Firstly, we investigated biochemical interaction by inhibiting STAT3 and β-catenin (through gene knock-down and dominant-negative TCF4 expression) in nine CRC cell lines. β-catenin inhibition did not affect STAT3 levels, whereas STAT3 knock-down resulted in reduced β-catenin mRNA and protein levels. The reduction in β-catenin protein was not prevented by proteasome inhibition, and IL6-induced STAT3 activation resulted in increased β-catenin mRNA. This suggests that STAT3 positively regulates β-catenin (at a transcriptional level) and evaluation of 44 CRCs by immunostaining supported this by showing an association between nuclear STAT3 expression and nuclear β-catenin (*P* = 0.022). We tested the functional interaction between STAT3 and Wnt signalling by knocking down STAT3 and β-catenin individually and in combination. Knock-down of β-catenin and STAT3 individually inhibited cell proliferation (*P* < 0. 001 for each) through G1 arrest. However, simultaneous knock-down of STAT3 and β-catenin had a significantly weaker effect than knock-down of β-catenin alone (*P* < 0.01). Knock-down of STAT3 and β-catenin, individually and together, inhibited cell motility (*P* < 0.001) without evidence of interaction. We conclude that STAT3 regulates β-catenin but β-catenin does not regulate STAT3. The STAT3/β-catenin interaction is complex but may reduce the proliferative activity of β-catenin possibly by taking β-catenin protein beyond the optimal level. This may indicate biological differences in tumours where both STAT3 and β-catenin are activated compared to those where only one is activated.

## Introduction

Colorectal cancer (CRC) represents one of the most common causes of cancer-related death around the world (Stewart & Kleihues 2003). The development of CRC is a paradigm of multistep carcinogenesis with tumours arising from normal epithelium as a consequence of the stepwise accumulation of mutations (Ilyas *et al*. 1999). The ‘order’ of mutation [first described in the Fearon and Vogelstein model (Fearon & Vogelstein 1990)] is generally well conserved allowing mutations to be categorized as ‘early’ or ‘late’ events. The selection of mutations is driven by Darwinian evolution with mutations which confer a selective advantage allowing clonal expansion to occur (Greaves & Maley 2012). However, a new mutation will interact with the antecedent mutations, and there may be functional overlap between different mutations. It is possible therefore that some mutations may become redundant due to either a functional redundancy with subsequent mutations or a change in growth restraints (known as ‘oncogene amnesia’). Conversely, some mutations – even if acquired early in the carcinogenetic process – may remain essential for tumour cell viability (known as ‘oncogene addiction’) (Felsher 2008).

The earliest event in the development of CRC is probably activation of the canonical Wnt signalling pathway. In around 80–90% of cases (Rowan *et al*. 2000), this occurs through loss-of-function mutation of the *APC* gene which results in failure of the β-catenin destruction complex and abnormal accumulation of β-catenin protein. Less frequently mutations in the β-catenin gene (*CTNNB1)* occur which result in a degradation-resistant but transcriptionally active protein (Ilyas *et al*. 1997). A variety of downstream targets of β-catenin protein (such as c-Myc, c-Jun, MMP7, Survivin) are activated and lead to tumour initiation (Wilson *et al*. 1997; He *et al*. 1998; Mann *et al*. 1999; Tetsu & McCormick 1999; Zhang *et al*. 2001; Conacci-Sorrell *et al*. 2002).

The signal transducer and activator of transcription 3 (STAT3) pathway is found to be activated in around 50–60% of CRCs (Morikawa *et al*. 2011). In contrast to Wnt signalling, however, activation of this pathway is usually considered a late event (Kusaba *et al*. 2005). Activation of STAT3 occurs following ligand binding to a variety of membranous receptors including receptors for cytokines, hormones and growth factors. However, various intracellular kinases (such as the src family kinases) can activate STAT3 without receptor involvement (Turkson *et al*. 1998; Bromberg *et al*. 1999; Simon *et al*. 2000). Activated STAT3 is usually phosphorylated on Tyrosine 705 (Bromberg *et al*. 1999) and, in this form, it translocates to the nucleus where it can act as a transcription factor. Amongst its targets are molecules involved in cell proliferation [such as Cyclin D1 and C-Myc (Yu *et al*. 2009)], apoptosis [such as BCL-2 and BCL-X (Yu *et al*. 2009)] and cell motility [such as TWIST (Cheng *et al*. 2008)].

Wnt and STAT3 signalling are activated at opposite ends of the adenoma–carcinoma sequence. That is to say that deregulation of Wnt signalling is thought to be the first step in the conversion of normal tissue to adenoma. In contrast, deregulation of STAT3 is thought to occur in late stage adenomas or invasive carcinomas (Kusaba *et al*. 2005). Given the difference in the timing of activation, it is probable that Wnt and STAT3 signalling confer different selective advantages to tumour cells. There is a degree of overlap in the reported downstream targets activated by Wnt signalling and STAT3 signalling. In addition, there is evidence that STAT3 and β-catenin may influence each other. Thus, in breast cancer, it has been reported that STAT3 upregulates the expression and function of β-catenin (Armanious *et al*. 2010) whilst in oesophageal and some haematological malignancies, β-catenin appears to upregulate the expression of STAT3 (Yan *et al*. 2008; Anand *et al*. 2010). In CRCs, published studies have generally examined the effect of STAT3 on β-catenin with one study claiming that STAT3 caused nuclear localization and increased transcriptional activity of β-catenin (Kawada *et al*. 2006). Another study reported that abrogation of STAT3 signalling causes uncoupling of β-catenin from E-Cadherin at the cell–cell junctions (thereby inducing cell motility), but it has no effect on transcriptional activity of β-catenin (Rivat *et al*. 2004).

In this study, we sought to elucidate the interactions of STAT3 and β-catenin at both the biochemical level and the biological level in CRC. Each gene was knocked down in several CRC cell lines, and the effects of this on the expression of the other gene and on the proliferation and motility of tumour cells were tested.

## Materials and methods

### Tissue culture

The human CRC cell lines SW620, SW480, HT29, HCT116, SW837 and SW948 were originally obtained from the American Type Culture Collection (ATCC). The human CRC cell lines SW1222, HT55 and C106 were originally obtained from the European Collection of Cell Cultures (ECACC). These cell lines were a kind gift from Prof Ian Tomlinson (Molecular and Population Genetics Laboratory, London Research Institute, Cancer Research UK, London). The identity of the cell lines was confirmed prior to commencing the work by mutation profiling as previously described (Fadhil *et al*. 2010). Cells were grown in Dulbecco's modified Eagle's medium (DMEM; Invitrogen Cell Culture, Paisley, UK) supplemented with 10% fetal bovine serum (FBS; Gibco Invitrogen Cell Culture, Paisley, UK), 100 U/ml penicillin and 100 μg/ml streptomycin and maintained at 37 °C and 5% CO2 in a humidified incubator.

### Cell transfection

STAT3 and β-catenin were knocked down using small interfering RNA (siRNA) as previously described (Albasri *et al*. 2011). For the knock-down of both, prevalidated Stealth siRNA duplexes (Invitrogen) were transfected into the cells. Two different duplexes were used for STAT3, and one was used for β-catenin; control cells were transfected with siRNA targeted to luciferase (sequences for all duplexes are given in Table S1). Cells were transfected at 30–50% confluence using lipofectamine 2000 (Invitrogen) to give a final siRNA concentration of 33 nM and were harvested 48 h after transfection. As an alternative to knock-down of β-catenin, inhibition of Wnt signalling was also undertaken by forced transfection of cells with dominant-negative TCF4 (DN-TCF4) protein. The DN-TCF4 protein expressed by the plasmid prevents β-catenin from binding to the promoter regions of β-catenin target genes and therefore prevents β-catenin-mediated transcriptional activation (Morin *et al*. 1997). The cell lines HCT116 and HT29 were transfected with DN-TCF4 expression vector (or empty vector controls) using lipofectamine 2000 as previously described (Ahmed *et al*. 2010), and levels of STAT3 protein were tested 72 h after transfection.

For experiments investigating the biological interaction of STAT3 and β-catenin, both genes were silenced independently and together in SW620 cells. For concomitant knock-down, each duplex was added at 33 nM making a final siRNA duplex concentration of 66 nM. In these experiments, the final duplex concentration was kept identical and thus, in conditions where only one of the genes was knocked down, the final 66 nM concentration was achieved by correction with luciferase specific siRNA.

### Quantitative Reverse Transcription PCR

Quantitative Reverse Transcription PCR (Q-RT-PCR) was carried out to quantify mRNA of both STAT3 and β-catenin. Total RNA was extracted from the cells using the RNeasy Mini Kit (Qiagen, Manchester, UK) following the manufacturer's protocol. cDNA was prepared with the SuperScript III Reverse Transcriptase kit (Invitrogen, Paisley, UK). PCR was performed in a total volume of 25 μl consisting of 1X Brilliant SYBR Green QPCR Master Mix (Stratagene, Cambridge, UK), primers (forward and reverse) at a final concentration of 250 nM, 30 nM ROX (reference dye) and 10 ng of the cDNA template. Samples were tested in triplicate, and no template control and non-reverse transcribed mRNA was included to detect any cDNA or DNA contamination. PCR was performed on an MX3005P QPCR machine (Stratagene) using single thermal cycling profile of (95 °C/10 min) X1; [(95 °C/30 s)/(56 °C/1 min)/(72 °C/1 min)] X40; (72 °C/10 min) X1. Quantification was by the standard curve method using MX3005P QPCR System Software version 4.01, and data were normalized to the HPRT housekeeping gene. The sequence of each primer pair and their products size are detailed in Table S2.

### Western blotting

Whole cell extracts were prepared using commercially available RIPA lysis buffer [20 mM Tris, pH 7.5, 150 mM NaCl, 1% TritonX-100, 0.5% sodium deoxycholate, 1 mM EDTA, 0.1% SDS, supplemented with protease and phosphatase inhibitors (Sigma, Gillingham, UK)]. Protein (40 μg in total) was loaded on a 10% SDS–PAGE gel and transferred on to PVDF membranes (Amersham Bioscience, Buckinghamshire, UK) by semidry transfer. After blocking for 1 h with 5% (w/v) milk powder dissolved in 0.1% TPBS (Tween 20 in Phophate Buffered Saline solution, Sigma), membranes were incubated overnight at room temperature with the indicated primary antibody. Antibodies and their dilutions were anti- β-actin (Sigma-Aldrich) at 1/2000 dilution, anti STAT3 (Abcam ab50761) at 1/100, anti- c-Myc (clone 9E10; Abcam, Cambridge, UK) at 1/500, anti-β-catenin (ALX-804-060-C100; Enzo Life Sciences, Exeter, UK) at 1/500. After three 10-min washes in 0.1% TPBS, membranes were incubated for 1 h at room temperature with the appropriate horseradish peroxidase-linked secondary antibody. Following three further washes, detection of bound antibody was performed using the enhanced chemiluminescence kit (Pierce, Rockford, IL, USA). Bands were visualized using X-ray films (Kodak, Hemel Hempstead, UK).

### Proteasome inhibition

To ascertain the role of proteasomal degradation in the STAT3-mediated regulation of β-catenin, the proteasome inhibitor Z-Leu-Leu-Phe-CHO (Sigma-Aldrich) dissolved in DMSO was added to SW620 cells at a final concentration of 2 μM. Cells were exposed to the proteasomal inhibitor 24 h after transfection with siRNA duplexes. To confirm the effectiveness of the proteasome inhibition (i.e. a positive control), the CRC cell line RKO was treated with the proteasome inhibitor and compared with control cells treated with an equivalent volume of DMSO carrier. The RKO cell line has an intact β-catenin degradation system making β-catenin undetectable by Western blot (da Costa *et al*. 1999). However, successful inhibition of proteasome activity results in β-catenin becoming detectable.

### Stimulation of the STAT3 pathway

Interleukin-6 can be used to stimulate STAT3 expression and activity (Yang & Stark 2008). Human recombinant IL-6 (Immunotools) was used to stimulate the STAT3 signalling pathway in SW620. Cell lines were stimulated with IL-6 at a concentration of 20 ng/ml for 12 h. RNA was extracted at the end of the incubation, and the effect of STAT3 stimulation was determined by Q-RT-PCR.

### Tumour sample collection and immunostaining

Local approval for tissue access and study was granted by the Nottingham Research Biobank (REC reference 05/Q1605/66). A total of 44 anonymized cases of sporadic CRC who underwent surgery between 2004 and 2005 were selected from the archives of the Nottingham University Hospitals Department of Histopathology. One tumour block from each case was selected, and a tissue microarray (TMA) was constructed as previously described (Ullenhag *et al*. 2007). For immunostaining, 4 μm-thick TMA sections were dewaxed with xylene, rehydrated through graded alcohol and then boiled in sodium citrate buffer (0.01 M, pH6) for 10 min for antigen retrieval. Endogenous peroxidase activity was blocked by immersion in a solution of 0.3% hydrogen peroxide (in water) for 15 min. Immunostaining was performed using the Dako REAL™ kit, and all steps were performed at room temperature. Sections were incubated for 1 h with primary mouse monoclonal antibody diluted in Dako REAL™ diluent buffer (STAT3 (ab50761; Abcam) at 1/20 dilution, β-catenin (M3539; Dako) at 1/200 dilution). Diluent buffer alone without the primary antibody served as a negative control. Bound antibody was detected using 3, 3′-Diaminobenzidine tetrahydrochloride as the chromogen (diluted 1:50 in Dako REAL™ HRP buffer) for 10 min. Sections were counterstained with haematoxylin. The immunostaining was evaluated separately by MI and SI, discrepant cases (where the score varied by more than 10%) were reviewed together, and a consensus score was reached. Cytoplasmic and membranous staining was evaluated using the well-described H-score system (Walker 2006), whilst nuclear staining was scored simply as percentage of tumour nuclei showing positive staining.

### Cell proliferation and apoptosis

The effect of gene silencing on cell number was evaluated at different time points by the methylene blue assay as previously described (Dvory-Sobol *et al*. 2007). Briefly, 48 h following transfection, cells were trypsinized, each well of a six well plate (Costar) was seeded with 5 × 10^4^ cells and the number of viable cells, and apoptotic bodies were measured at specific time points of 24, 48 and 72 h. Apoptosis was measured by manually counting trypan blue positive cells in the overall cell population. Cells were trypsinized 72 h after transfection, resuspended in serum free medium, stained with trypan blue and counted under the microscope using haemocytometer. The percentage of apoptotic cells was calculated as the stained cells (i.e. apoptotic cells) divided by the total. Each experiment was performed in triplicate and repeated on at least three separate occasions.

### Cell cycle analysis

The effect of gene silencing on the cell cycle was evaluated by flow cytometry. Briefly, the transfected cells were grown in 24 well plates for 48 h before harvesting. Cells were rinsed with PBS and then fixed with 70% ice-cold ethanol for 30 min. Following two washes with 0.1% foetal serum albumin in PBS, the cells were suspended in 1 ml 0.1% FBS containing 10 μl RNase solution (10 mg/ml, DNAse free; Sigma) and 50 μl propidium iodide solution (1 mg/ml; Sigma) for 20 min before being analysed on a Beckman Coulter flow cytometer (Beckman Coulter, Brea, CA, USA). A minimum of 3 × 10^4^ events were captured, and the data were analysed by of Weasel software version 2.7 (Walter and Elisa Hall Institute in Australia). This assay was performed in triplicate on three separate occasions.

### Cell migration

Transwell cell migration was measured using a Boyden chamber containing a polycarbonate filter with an 8 μm pore size (Costar). Cells were serum starved for synchronization, and 5 × 10^4^ cells were seeded in the top chamber in medium containing 10% foetal calf serum. The lower chamber contained medium which was enriched with 20% foetal calf serum. Migration across the membrane was assessed after 48 h by fixing the cells attached to the bottom surface in 10% methanol for 30 min. These were then stained for 30 min with methylene blue, and the stained cells manually counted.

### Statistical analysis

Statistical analysis was performed using the GraphPad-Prism program (GraphPad Software Inc., La Jolla, CA, USA). An unpaired *t*-test was used to evaluate data derived from the studies of proliferation, apoptosis and cell motility. A Fisher's exact test was used to test for the association between the expression of nuclear STAT3 and nuclear β-catenin. All tests were two tailed, and a *P*-value of <0.05 was taken as statistically significant.

## Results

### STAT3 is a positive regulator of β-catenin protein

**Figure 1:**
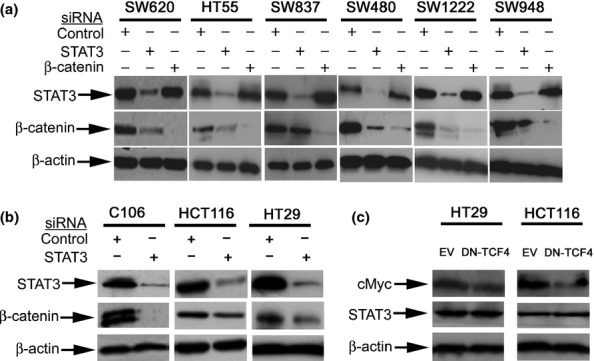
Western blot data showing that knock-down of STAT3 results in downregulation of β-catenin but not vice versa. (a) This was a reproducible effect seen in six different colorectal cancer cell lines. (b) This shows the effect of STAT3 knock-down, when tested in a further three colorectal cancer cell lines, was a reduction in β-catenin levels. (c) shows that forced expression of dominant-negative TCF4 (DN-TCF4) caused downregulation of c-Myc but had no effect on STAT3 when compared to empty vector (EV).

Given the mixed published data regarding the nature of the biochemical interaction of STAT3 and β-catenin, we first sought to ascertain whether STAT3 and β-catenin were able to regulate each other. Initially, STAT3 and β-catenin were knocked down individually in six different CRC cell lines that is SW620, HT55, SW837, SW480, SW1222 and SW948, and the effect on protein levels was tested. There was some variation in the level of protein knock-down between cell lines which reflects the variation in transfection efficiency. However, there was robust knock-down of both proteins with the siRNAs used. In every cell line, when β-catenin was knocked down, there was no associated change in the level of STAT3 protein. In contrast, when STAT3 was knocked down, this was associated with a marked reduction in the level of β-catenin protein (Figure1). We extended our study by knocking down STAT3 only in a further three cell lines that is HT29, C106 and HCT116 and, our observations were identical (Figure1b). As there was no effect on STAT3 levels by β-catenin, the experiments of knock-down of β-catenin were repeated. As ‘off target’ effects are a classical confounder with gene knock-down, we knocked down STAT3 using a different siRNA duplex in three cell lines (i.e. HCT116, HT29 and SW620) and observed the same effect on β-catenin protein (Figure S1). As an alternative method to inhibiting Wnt signalling, we transfected HT29 and HCT116 with DN-TCF4 expression constructs. Forced expression of DN-TCF4 caused a reduction in c-Myc protein (a well-described target of Wnt signalling) but had no effect on STAT3 levels (Figure1c). Thus, our data from a total of nine cell lines suggest that STAT3 regulates β-catenin levels but not vice versa.

**Figure 2:**
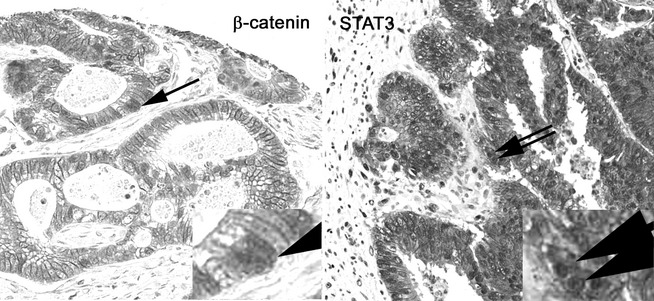
Immunohistochemical expression of β-catenin and STAT3. The staining for β-catenin (left panel) was seen in membranous, cytoplasmic and nuclear compartments. The expression of STAT3 was in the cytoplasm and the nucleus. There was a significant association between nuclear expression of β-catenin (arrow) and STAT3 (double arrow). High-power inserts shown in the corners for both proteins.

The immunohistochemical expression of STAT3 and β-catenin was tested in a small series of primary CRCs. Staining for β-catenin was seen in all compartments (i.e. membranous, cytoplasmic and nuclear), whilst STAT3 staining was seen only in the cytoplasm and nuclei. The mean score for all assessable cores from each individual case was used in analysis. For analysis of cytoplasmic staining (of both proteins), the median score was used as a cut-off point to dichotomize into ‘positive’ and ‘negative’. For nuclear staining for STAT3, the median score was used as the cut-off whilst, for β-catenin, any nuclear staining was scored as positive. There was a positive association between nuclear STAT3 expression and nuclear β-catenin expression (*P* = 0.022, Table1, Figures2 and S2).

**Table 1:** The results of the immunostaining for STAT3 and β-catenin in a series of 44 primary colorectal tumours. A significant positive association (*P* = 0.022) was found between nuclear expression of these two proteins

	STAT3 +	STAT3−	Total
β-catenin +	10	8	18
β-catenin −	5	21	26
Total	15	29	44

### STAT3 alters β-catenin protein through transcriptional regulation

**Figure 3:**
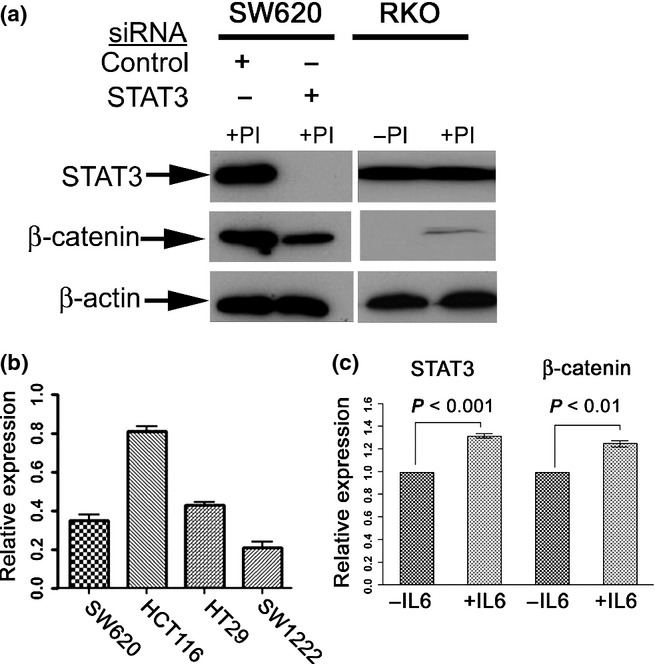
Regulation of β-catenin by STAT3 is probably at the transcriptional level: (a) shows that exposure of SW620 to the proteasome inhibitor (PI) Z-Leu-Leu-Phe-CHO did not prevent the downregulation of β-catenin following STAT3 knock-down. The cell line RKO was used to confirm the efficacy of proteasome inhibition as exposure to PI resulted in the appearance of a visible band for β-catenin. +PI indicates cells were exposed to PI and –PI indicates controls exposed to DMSO carrier. (b) shows that knock-down of STAT3 resulted in a downregulation of β-catenin mRNA (data shown as relative expression compared to control siRNA). (c) demonstrates stimulation of cells with IL-6 (as an alternative method of activating STAT3) resulted in upregulation of both STAT3 mRNA and of β-catenin mRNA (data shown as relative expression compared to control vehicle).

Changes in β-catenin protein can occur either through altered protein stability or through altered transcription. To explore the mechanism of STAT3 mediated regulation of β-catenin, we knocked down STAT3 in SW620 and exposed the cells to a proteasome inhibitor. This did not prevent the reduction in the level of β-catenin suggesting that inhibition of proteasome-mediated degradation was not the mechanism through which STAT3 regulated β-catenin (Figure3a). The functional activity of the proteasome inhibitor was confirmed in RKO; these cells do not usually have detectable β-catenin protein but, on exposure to the proteasomal inhibitor, a β-catenin band was visible on Western blot.

To test whether STAT3 altered β-catenin transcription, we measured β-catenin mRNA levels in four cell lines following STAT3 knock-down. In all four cell lines, a fall in STAT3 levels was accompanied by a fall in β-catenin mRNA levels (Figure3b). To further investigate this, we tested the effect of IL-6 stimulation of SW620. Stimulation with IL-6 is known to activate STAT3 signalling and induce STAT3 expression(Brocke-Heidrich *et al*. 2004). Our data showed that IL-6 stimulation of SW620 resulted in upregulation of both STAT3 and β-catenin mRNA levels (Figure3c, *P* < 0.001 and *P* < 0.01 respectively).

### STAT3 and β-catenin have a complex biological interaction

Having shown that STAT3 can regulate β-catenin, we next sought to ascertain the biological significance of this interaction. STAT3 and β-catenin were knocked down individually and in combination in SW620 to produce four different conditions that is control cells exposed to luciferase siRNA (SW620^control^), STAT3 knock-down only (SW620^STAT3-^), β-catenin knock-down only (SW620^βcat−^) and knock-down of both STAT3 and β-catenin (SW620^STAT3−^/^β-cat−^). The effect of each of these conditions was tested in a variety of functional assays.

**Figure 4:**
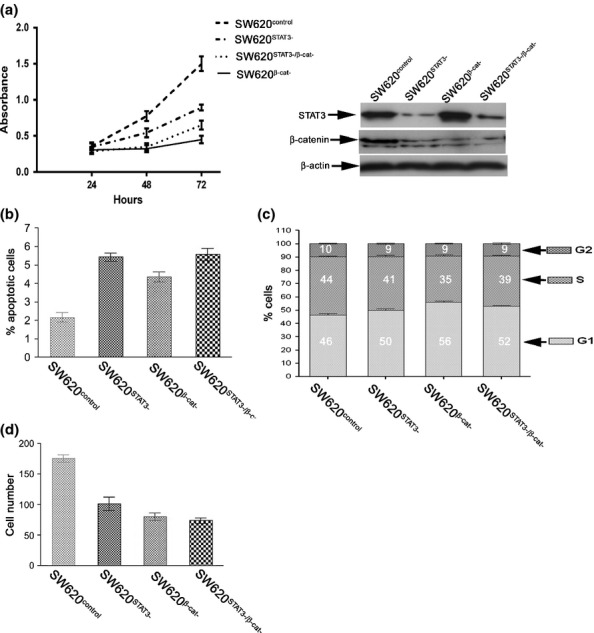
STAT3 and β-catenin interact in a complex manner. (a) shows that individual knock-down of both STAT3 and β-catenin (SW620^STAT^^3−^ and SW620^βcat−^ respectively) results in inhibition of cell proliferation when compared with controls (SW620^control^). β-catenin has a more marked effect although combined knock-down (SW620^STAT^^3−^/^β-cat−^) results in a lesser effect than knock-down of β-catenin alone (*P* < 0.01). The right hand panel confirms knock-down of STAT3 and β-catenin. (b) shows that there was no difference in the percentage of apoptotic cells. (c) demonstrates flow cytometry analysis of SW620 following knock-down of STAT3 and β-catenin. This shows that in all conditions, there are changes in the proportion of cells in G1 phase and that delayed progression through G1 may be the partial loss of β-catenin function that is exerted by STAT3. The numbers in the boxes represent the percentage of cells in each phase of the cell cycle. (d) STAT3 and β-catenin both inhibit cell motility but there is no notable interaction when they are knocked down in combination compared to individual knock-down.

Evaluation of cell numbers over 3 days showed that, compared to controls, each knock-down condition resulted in a fall in cell number (Figure4a). STAT3 knock-down alone caused a reduction in cell number by 30% (SW620^STAT3−^ vs. SW620^control^, *P *<* *0.001), and β-catenin knock-down alone only caused a reduction in cell number by 56% (SW620^βcat−^ vs. SW620^control^, *P *<* *0.001). However, combined knock-down caused a reduction in cell number of 45% which was significantly less than that caused by β-catenin knock-down alone (SW620^STAT3−^/^β-cat−^ vs. SW620^βcat−^, *P *<* *0.01). These findings were reflected to some degree (albeit without a statistically significant effect) when apoptotic cells were evaluated. Numerically, the percentage of apoptotic cells was SW620^control^ = 2%, SW620^STAT3−^= 5.4%, SW620^βcat−^ = 4.4% and SW620^STAT3−^/^β-cat−^ =5.7% (Figure4b).

To further investigate the unexpected effects of gene knock-down on cell number, each of the four conditions underwent cell cycle analysis by flow cytometry (Figure4c). STAT3 knock-down resulted in a greater number of cells in G1 compared to control cells (SW620^STAT3−^ vs. SW620^control^, 50% vs. 46% respectively). As anticipated, β-catenin knock-down alone resulted in a greater proportion of cells in G1 (SW620^βcat−^ = 56%) but this was greater than the combined knock-down (SW620^STAT3−^/^β-cat−^ = 52% in G1). In all knock-down conditions, the increase in the cells in G1 phase was accompanied by equivalent reduction in the percentage of the cells in S phase with relatively little change in G2.

As both STAT3 and β-catenin can influence cell motility, we tested their interaction on cell motility using the transwell assay. In each condition (SW620^STAT3−^, SW620^βcat−^ and SW620^STAT3−^/^β-cat−^), there was a significant reduction in cell motility compared to the control (*P* < 0.001 for each, Figure4d). There was some variation between each of the conditions, but this was not statistically significant.

## Discussion

In the colon, activation of the Wnt signalling pathway is usually an ‘early’ event involved in tumour initiation, whilst activation of STAT3 signalling is considered a ‘late’ event involved in tumour invasion and metastasis. However, there is a degree of functional redundancy in these pathways, and it is likely that they will interact with each other in some way. Our data show that, in contrast to studies conducted in oesophageal cancer (Yan *et al*. 2008) and anaplastic large cell lymphoma (Anand *et al*. 2010), β-catenin does not influence levels of STAT3 protein in CRC cell lines. However, in experiments conducted in nine different CRC cell lines and using two different siRNA duplexes, we were able to demonstrate that knock-down of STAT3 was associated with a reduction in the level of β-catenin protein. This suggests that STAT3 is a positive regulator of β-catenin and is supported by our analysis of primary tumours showing a positive association between nuclear β-catenin and nuclear STAT3 expression. Our data run contrary to those of Kawada *et al*. (Kawada *et al*. 2006) who, in the CRC cell line SW480, were not able to demonstrate a change in β-catenin protein following inhibition with dominant-negative STAT3. Kawada *et al*. claimed that the effect of STAT3 was to promote nuclear localization of β-catenin protein (with consequent transcriptional activation of targets) but they found no effect of STAT3 on E-cadherin/β-catenin interaction at the cell membrane. These findings are directly contradicted by Rivat *et al*. (2004) who found that STAT3 had no effect on either nuclear localization or transcriptional activity of β-catenin but that a dominant-negative STAT3 caused uncoupling of the membranous E-cadherin-β-catenin complex. Although it is not stated, the images shown in Rivat's paper are highly suggestive of a reduction in total of β-catenin protein level. Thus our data, taken together with those seen in Rivat's paper, would suggest that STAT3 is a positive regulator of β-catenin protein.

We sought to ascertain the mechanism by which STAT3 could alter β-catenin protein levels. We found that knock-down of STAT3 was associated with a fall in β-catenin mRNA levels and that an increase in β-catenin mRNA levels was observed following stimulation of cells with IL-6 (a known activator of STAT3). The fall in β-catenin protein level following STAT3 knock-down could not, however, be prevented by a proteasome inhibitor. This would suggest that β-catenin is transcriptionally regulated by STAT3 and is consistent with studies in breast cancer showing direct binding of STAT3 to the β-catenin promoter (Armanious *et al*. 2010).

We next investigated the biological interaction between the STAT3 and Wnt signalling pathways. Co-operation of β-catenin with other oncogenes is well described (Janssen *et al*. 2006; Mologni *et al*. 2010) although it is particularly interesting in this case as we have shown that STAT3 signalling can alter levels of β-catenin protein. We knocked down STAT3 and β-catenin protein individually and in combination. We found that, in agreement with Kawada *et al*., inhibition of STAT3 resulted in a reduction in cell numbers. Knock-down of β-catenin resulted in a more pronounced reduction of cell numbers. However, combined knock-down resulted in a significantly less pronounced reduction than knock-down of β-catenin alone (*P* < 0.01). This would suggest that, either directly or indirectly, STAT3 has an inhibitory effect on β-catenin-driven proliferative activity. Such an effect has also been described in mouse models where loss of STAT3 promoted proliferation in germline Apc mutant Min mice (Musteanu *et al*. 2009). It is generally regarded that, for optimal activity, β-catenin protein levels must operate at ‘just right’ levels (Albuquerque *et al*. 2002). We would speculate that increases in β-catenin caused by STAT3 could take protein levels beyond the optimal range and thereby compromise β-catenin function.

Evaluation of apoptosis (through uptake of trypan blue) showed no difference between β-catenin and combine STAT3/β-catenin knock-down. However, cell cycle analysis raised the possibility that the inhibitory effect of STAT3 may be on progress through G1. Evaluation of cell motility showed more or less an equivalence between all three knock-down conditions with no evidence of interaction. This contradicts the data of Rivat *et al*. who found that dominant-negative STAT3 promoted invasive properties in cells.

Our data suggest that interaction between STAT3 and β-catenin results in a partial inhibition of cell proliferation but does not affect cell motility. It is highly likely that STAT3 will give a selective advantage, and as STAT3 activation is a late event, one can conjecture that a slight loss of proliferative activity is an acceptable cost for acquisition of other features which may be more relevant to the tumour at that stage. The biological interaction between STAT3 and β-catenin is obviously complex, but it raises the intriguing possibility that tumours in which both these pathways are activated may be biologically different to tumours in which in only of these pathways is activated. Such differences could be exploited to develop further personalized medicine for CRC.

In summary, we have investigated the biochemical and biological interaction of the Wnt and STAT3 signalling pathways in CRC. We have shown that STAT3 can regulate β-catenin but not vice versa. Furthermore, the biological interaction between these two pathways is complex, and it is possible that, in advanced CRCs, STAT3 is partially inhibiting β-catenin-driven proliferation.

## Supporting Information

**Figure S1.** This shows the effect of STAT3 knock-down with a different siRNA duplex (denoted as STAT3(ii)). There was a reduction in β-catenin levels when tested in three colorectal cancer cell lines.

**Table S1.**This shows the sequences for the siRNA duplexes used in the study. **Table S2.** This shows the sequences for the primers used for PCR in the study.
