# Supplementary figures and images for: STAT3 paradoxically stimulates β-catenin expression but inhibits β-catenin function
Source: Int J Exp Pathol. 2014 Oct 28;95(6):392–400. doi: 10.1111/iep.12102 (PMC4285465; doi:10.1111/iep.12102)

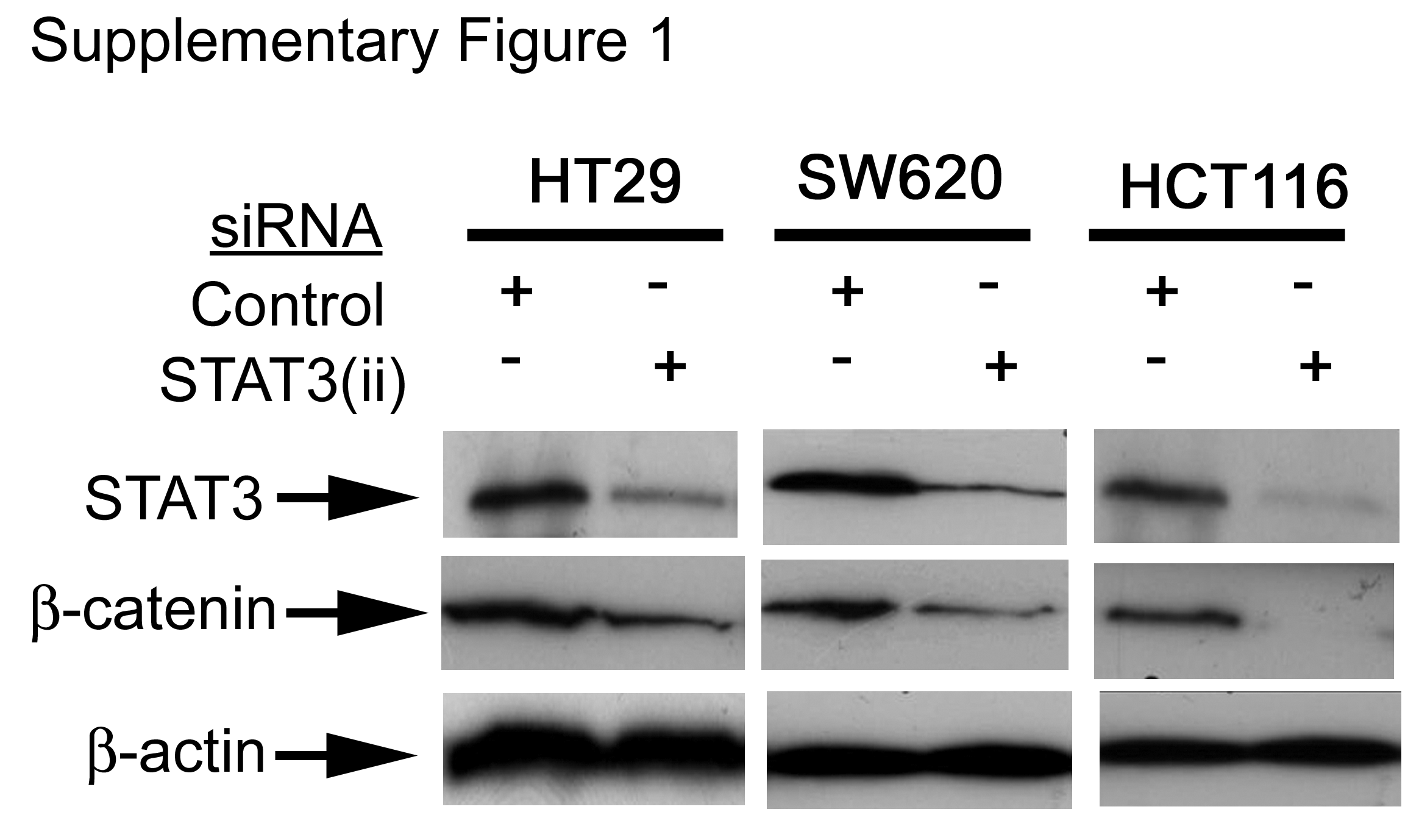

Supplement: Supplementary file 1 — Figure S1. This shows the effect of STAT3 knock-down with a different siRNA duplex (denoted as STAT3(ii)). There was a reduction in β-catenin levels when tested in three colorectal cancer cell lines. [file iep0095-0392-sd1.tif]
