# Supplementary material for: STAT3 paradoxically stimulates β-catenin expression but inhibits β-catenin function
Source: Int J Exp Pathol. 2014 Oct 28;95(6):392–400. doi: 10.1111/iep.12102 (PMC4285465; doi:10.1111/iep.12102)
Supplement: Supplementary file 2 — Table S1.This shows the sequences for the siRNA duplexes used in the study. Table S2. This shows the sequences for the primers used for PCR in the study. [file iep0095-0392-sd2.docx]

**Supplementary Table 1**

| siRNA -STAT3-1 | 5’-UGCUGUAGCUGAUUCCAUUGGGCCA |
| --- | --- |
| siRNA -STAT3-2 | 5’-GCCAAUUGUGAUGCUUCC |
| siRNA-b-cat | 5’-GGCCUGGUUUGAUACUGACCUGUA A. |
| siRNA-luciferase | 5’-CAGUGUAGUAGUCGUUUCUCGGAUA |

Supplementary Table 2. Primer sequences.

| **The gene** | **Forward primer (5’→3’)** | **Reverse primer (5’→ 3’)** | **Amplicon Size(bp)** |
| --- | --- | --- | --- |
| **HPRT**  [*NM_000194.2*](http://www.ncbi.nlm.nih.gov/entrez/viewer.fcgi?db=nucleotide&id=164518913) | AAATTCTTTGCTGACCTGCTG | TCCCCTGTTGACTGGTCATT | 122 |
| **STAT3**  [*NM_003150*](http://www.ncbi.nlm.nih.gov/entrez/viewer.fcgi?db=nucleotide&id=164518913) | AGTTTCTGGCCCCTTGGATT | AAGCGGCTATACTGCTGGTC | 118 |
| **β-Catenin**  [*NM_001098209.1*](http://www.ncbi.nlm.nih.gov/entrez/viewer.fcgi?db=nucleotide&id=148233337) | GACCACAAGCAGAGTGCTGA | TGCAGCATCTGAAAGATTCCT | 163 |
| **C-Myc**  [*NM_002467.4*](http://www.ncbi.nlm.nih.gov/nuccore/NM_002467.4) | TTCGGGTAGTGGAAAACCAG | CAGCAGCTCGAATTTCTTCC | 203 |
